# Transcranial alternating current stimulation improves quality of life in Parkinson’s disease: study protocol for a randomized, double-blind, controlled trial

**DOI:** 10.1186/s13063-024-08045-5

**Published:** 2024-03-20

**Authors:** Hong-yu Zhang, Ting-ting Hou, Zhao-hui Jin, Tian Zhang, Yi-heng Wang, Zi-hao Cheng, Yong-hong Liu, Jin-ping Fang, Hong-jiao Yan, Yi Zhen, Xia An, Jia Du, Ke-ke Chen, Zhen-zhen Li, Qing Li, Qi-ping Wen, Bo-yan Fang

**Affiliations:** 1 Parkinson Medical Center, Beijing Rehabilitation Hospital, Capital Medical University, Badachu, Xixiazhuang, Shijingshan District, Bejing, 100144 China; 2 Capital Medical University, Beijing, China; 3 Radiology Department, Beijing Rehabilitation Hospital, Capital Medical University, Badachu, Xixiazhuang, Shijingshan District, Bejing, 100144 China

**Keywords:** Transcranial alternating current stimulation, Parkinson’ s disease, Multidisciplinary intensive rehabilitation treatment, Randomized controlled trial

## Abstract

**Background:**

The neural cells in the brains of patients with Parkinson’s disease (PWP) display aberrant synchronized oscillatory activity within the beta frequency range. Additionally, enhanced gamma oscillations may serve as a compensatory mechanism for motor inhibition mediated by beta activity and also reinstate plasticity in the primary motor cortex affected by Parkinson’s disease. Transcranial alternating current stimulation (tACS) can synchronize endogenous oscillations with exogenous rhythms, thereby modulating cortical activity. The objective of this study is to investigate whether the addition of tACS to multidisciplinary intensive rehabilitation treatment (MIRT) can improve symptoms of PWP so as to enhance the quality of life in individuals with Parkinson’s disease based on the central-peripheral-central theory.

**Methods:**

The present study was a randomized, double-blind trial that enrolled 60 individuals with Parkinson’s disease aged between 45 and 70 years, who had Hoehn-Yahr scale scores ranging from 1 to 3. Participants were randomly assigned in a 1:1 ratio to either the tACS + MIRT group or the sham-tACS + MIRT group. The trial consisted of a two-week double-blind treatment period followed by a 24-week follow-up period, resulting in a total duration of twenty-six weeks. The primary outcome measured the change in PDQ-39 scores from baseline (T0) to 4 weeks (T2), 12 weeks (T3), and 24 weeks (T4) after completion of the intervention. The secondary outcome assessed changes in MDS-UPDRS III scores at T0, the end of intervention (T1), T2, T3, and T4. Additional clinical assessments and mechanistic studies were conducted as tertiary outcomes.

**Discussion:**

The objective of this study is to demonstrate that tACS can enhance overall functionality and improve quality of life in PWP, based on the framework of MIRT. Additionally, it seeks to establish a potential correlation between these therapeutic effects and neuroplasticity alterations in relevant brain regions. The efficacy of tACS will be assessed during the follow-up period in order to optimize neuroplasticity and enhance its potential impact on rehabilitation efficiency for PWP.

**Trial registration:**

Chinese Clinical Trial Registry ChiCTR2300071969. Registered on 30 May 2023.

**Supplementary Information:**

The online version contains supplementary material available at 10.1186/s13063-024-08045-5.

## Administrative information

Note: the numbers in curly brackets in this protocol refer to the SPIRIT checklist item numbers. The order of the items has been modified to group similar items (see https://www.equator-network.org/reporting-guidelines/spirit-2013-statement-defining-standard-protocol-itemsfor-clinical-trials/).

**Table:** 

**Title {1}**	Transcranial alternating current stimulation improves quality of life in Parkinson’s disease: study protocol for a randomized, double-blind, controlled trial
**Trial registration {2a and 2b}**	Chinese Clinical Trial Registry ChiCTR2300071969. Registered on 30 May 2023. Statement: all items can be found within the protocol.
**Protocol version {3}**	Version 2.0 of September 11, 2023.
**Funding {4}**	The National Key R&D Program of China (Grant Number: 2022YFC3602603); Beijing Shijingshan District Medical Key Discipline Research Program (2023002)
**Author details {5a}**	Hong-yu Zhang and Ting-ting Hou were co-first authors. HYZ, TTH, TZ, YHW, ZHC: Capital Medical University, Beijing, China. BYF, ZHJ, YHL, JPF, HJY, YZ, XA, JD, KKC, ZZL, QL: Parkinson Medical Center, Beijing Rehabilitation Hospital, Capital Medical University, Badachu, Xixiazhuang, Shijingshan District, Bejing 100,144, China. QPW: Radiology Department, Beijing Rehabilitation Hospital, Capital Medical University, Badachu, Xixiazhuang, Shijingshan District, Bejing 100,144, China
**Name and contact information for the trial sponsor {5b}**	Bo-yan Fang, M.D. Email: fangboyanv@ccmu.edu.cn ORCID: Bo-yan Fang, 0000–0002-9935-433X
**Role of sponsor {5c}**	The funding body had no role in protocol design, statistical analysis and manuscript preparation.

## Introduction

### Background and rationale {6a}

Parkinson’s disease (PD) is a common neurodegenerative disease whose clinical manifestations include motor and non-motor symptoms [1]. Motor symptoms comprise resting tremor, bradykinesia, gait disturbance, and myotonia. Non-motor symptoms encompass cognitive dysfunction, sensory abnormalities, depression, anxiety, and autonomic dysfunction [2–4]. These symptoms have a significant impact on the quality of life of patients and result in substantial medical expenses [5, 6]. Neuronal oscillations in the brain play a key role in the pathophysiology of PD. Specific frequency patterns can control communication and network resonance between brain regions, and rapid pathological neuronal synchronization in PD may be a causative factor for motor and non-motor disorders [7, 8]. Abnormal synchronized beta oscillatory activity has been observed at multiple levels within the basal ganglia network in both people with Parkinson’s disease and animal models [9, 10]. The beta activity is associated with symptoms of Parkinson’s disease, such as rigidity and bradykinesia, and can be modulated by medication and deep brain stimulation (DBS). Suppression of beta power is also correlated with improvement in dyskinesia. In humans undergoing invasive recordings, both levodopa and therapeutic DBS have demonstrated the ability to decrease oscillatory synchronization within the beta range (13–30 Hz), while simultaneously promoting oscillatory synchronization within the gamma range (60–90 Hz) across multiple structures within the motor network [11–14]. The gamma band activity is generally considered to have a prokinetic effect, serving as a compensatory mechanism for the akinetic role played by beta activity [15, 16]. Increased gamma power has been observed at the initiation of exercise [17], during exercise [18], and during motor imagery [19]. Studies have demonstrated that cortical gamma oscillations play a beneficial role in modulating the long-term potentiation-like plasticity of M1 in Parkinson’s disease and improve motor symptoms in PWP [20, 21].

Transcranial alternating current stimulation (tACS), a non-invasive stimulation technique, is capable of modulating cortical activity by manipulating and entraining intrinsic oscillations through the injection of sinusoidal alternating current from the scalp, with the endogenous oscillations during entrainment synchronized with the extrinsic rhythmic stimulation [22, 23]. The extracellular application of alternating current (AC) electric field in tACS modulates neuronal transmembrane potentials at the cellular level, inducing sinusoidal changes. Even small polarizations of the cell membrane can significantly impact the firing rate of active neurons, while AC stimulation effects can be further amplified by neuronal networks [24, 25]. In recent years, mounting evidence suggests that tACS can modulate cortical rhythms by entraining endogenous oscillations and promote neuroplasticity in multiple brain regions, thereby enhancing motor function and alleviating non-motor symptoms in individuals with PWP [21, 26–29]. For security reasons, previous studies on the application of tACS in PWP have primarily focused on utilizing weak currents below 2 mA. Although these studies have demonstrated some efficacy in improving bradykinesia, tremor, and cognition among PWP [27, 30, 31], they have generally employed low energy levels and limited cortical region stimulation through single stimuli. Furthermore, the conclusions regarding long-term effects have been inconsistent [32]. Several recent studies have shown that high-intensity transcranial alternating current stimulation (Hi-tACS) with parameters of 77.5 Hz (gamma band) and 15 mA may be more potent inducers of certain deep brain regions and have been shown to be safe. The delivery of a current with a frequency of 77.5 Hz through electrodes placed on the forehead and mastoid areas has been reported to modulate beta-endorphin and neurotransmitter levels in the cerebrospinal fluid, brainstem, hypothalamus, and cortex [33, 34]. It has demonstrated efficacy in ameliorating chronic primary insomnia, refractory epilepsy, as well as anxiety and depression, while maintaining a proven safety profile [35–37]. However, the effectiveness and neurobiological mechanisms of Hi-tACS in improving motor and non-motor functions in PWP have not been investigated. It is crucial to explore the role of Hi-tACS as a large alternating current in Parkinson’s disease in terms of motor and non-motor symptoms, and quality of life.

The “central-peripheral-central” closed-loop rehabilitation theory is a comprehensive framework for rehabilitation, wherein central neuroplasticity activation facilitates the formation of a feedback loop to enhance or restore damaged neural pathways [38]. By stimulating the central nervous system, neuroplasticity can be promoted to improve or reestablish impaired neural connections. Additionally, peripheral nervous system activation enhances central nervous system control by transmitting sensory information, activating muscles, and improving blood circulation. This integrated approach aims to achieve overall rehabilitation effects. Multidisciplinary intensive rehabilitation therapy (MIRT) is a comprehensive program for PWP that encompasses various disciplines such as aerobic exercise and motor cognitive therapy. It emphasizes combining balance exercises with cognitively demanding motor tasks (dual-tasking) [39–43].

Therefore, our hypothesis is that the combination of MIRT with tACS could potentially augment the management of motor and non-motor symptoms in PWP and optimize the efficiency and efficacy of rehabilitation therapy, thereby ultimately improving the overall quality of life for individuals affected by PD. The effectiveness of tACS in treating PWP has been validated through a randomized controlled trial (RCT) involving both true and sham stimulation groups. Additionally, we have investigated the neural mechanism underlying tACS-induced amelioration of Parkinson’s disease symptoms using behavioral indices, neuroelectrophysiology, functional magnetic resonance imaging (fMRI), and biomarkers. This study aims to establish a theoretical foundation for developing an innovative rehabilitation treatment plan for Parkinson’s disease while implementing tACS stimulation protocols.

### Objectives {7}

#### Primary objective

To investigate the impact of tACS on improving the quality of life in PWP.

#### Secondary objective

1. To investigate the effect of tACS on motor and non-motor symptoms in PWP.
2. To establish a correlation between the amelioration of behavioral outcomes and alterations in functional connectivity of neural networks, as well as biomarkers indicative of neuroplasticity, while investigating the underlying mechanisms of neuroplasticity associated with rehabilitation interventions.

### Trial design {8}

**Fig. 1:**
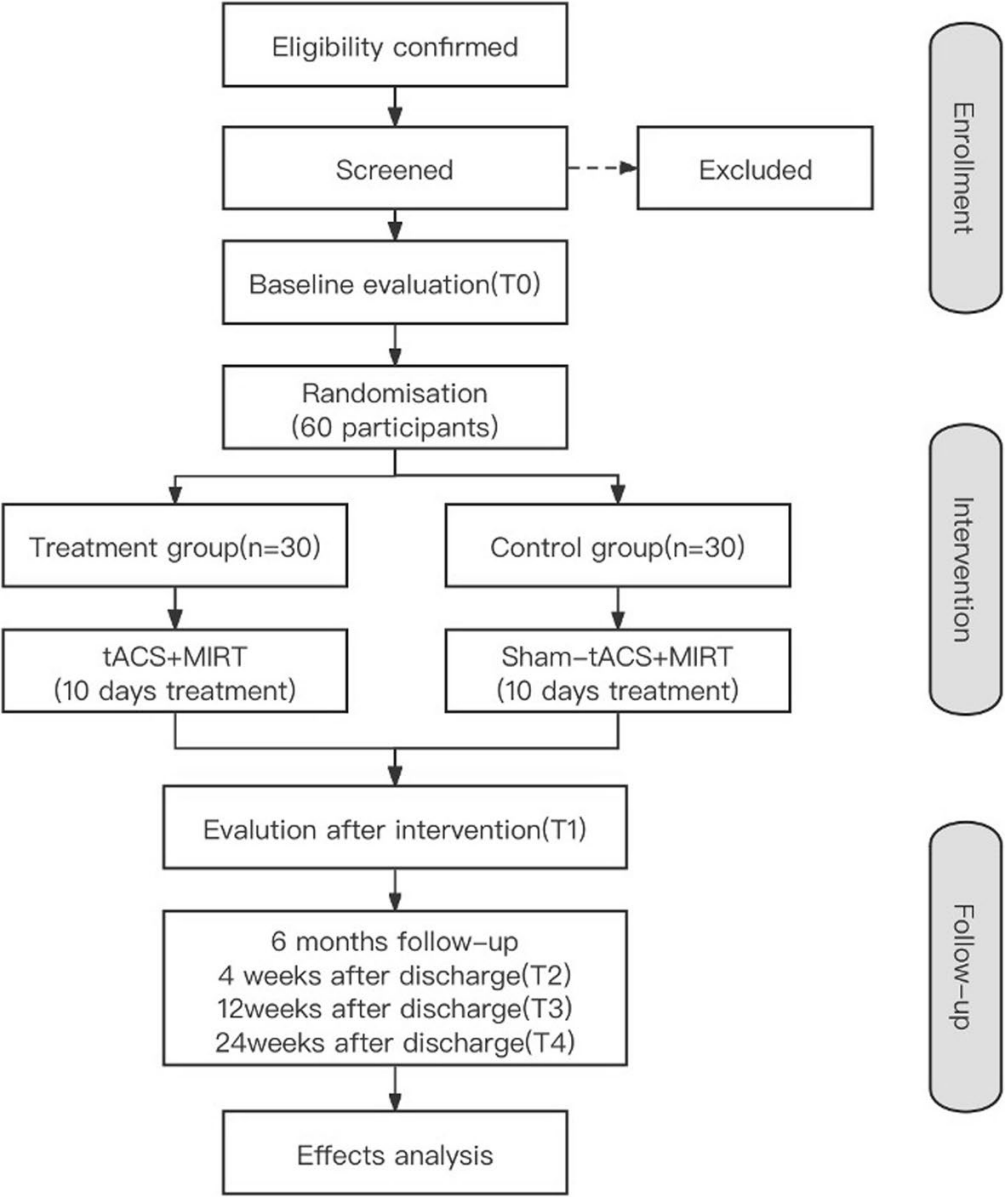
Flow chart of the experimental design

**Fig. 2:**
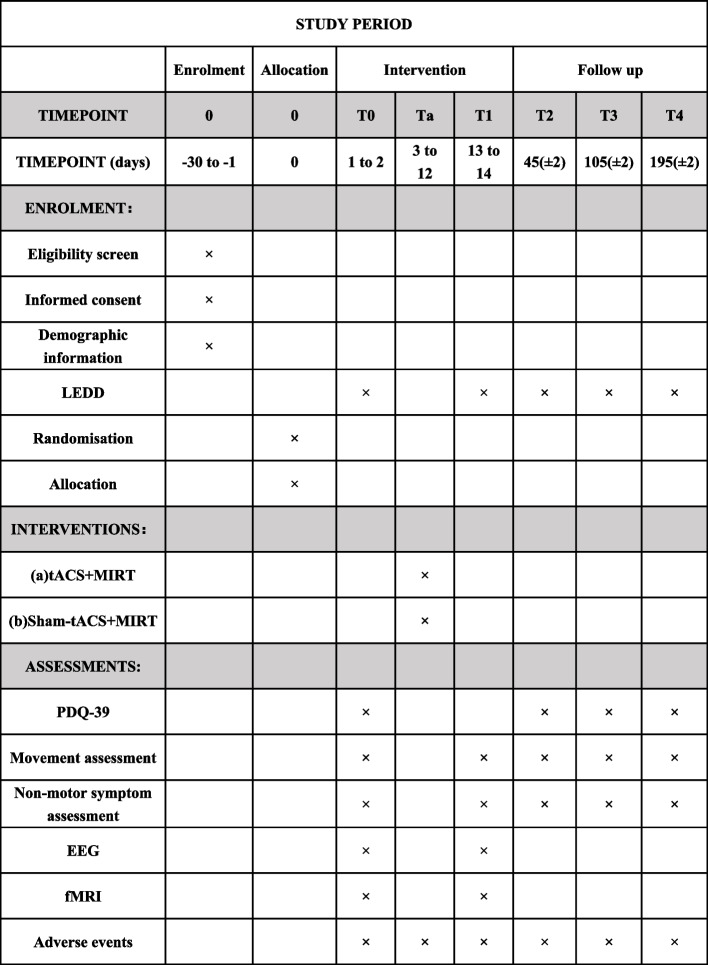
Schedule of enrolment, interventions, and assessments in accordance with the SPIRIT 2013 guidelines. LEDD, levodopa equivalent daily dose; tACS, transcranial alternating current stimulation; MIRT, multidisciplinary intensive rehabilitation treatment; PDQ-39, 39-item Parkinson’s Disease Questionnaire; EEG, electroencephalogram; fMRI, functional magnetic resonance imaging

The study will be a double-blind, randomized, placebo-controlled trial. The present protocol follows the Standard Protocol Items Recommendations for Interventional Trials (SPIRIT) guidelines and fulfills the SPIRIT checklist [44]. Our hypothesis is that tACS combined with MIRT is superior in terms of efficiency and effectiveness compared to MIRT alone for treating PD. Each patient’s participation in the study will last for 26 weeks, including baseline evaluation (T0), a 10-day intervention period, and assessments at the end of intervention (T1), as well as 4 weeks (T2), 12 weeks (T3), and 24 weeks (T4) post-intervention. Please refer to Fig. 1 for details regarding patient visits and follow-up schedule. Additionally, Fig. 2 provides an overview of all interventions and outcome measures in accordance with SPIRIT 2013 guidelines [45] (Additional file 1).

## Methods: participants, interventions, and outcomes

### Study setting {9}

This study will be conducted at a single site: Beijing Rehabilitation Hospital, Capital Medical University. The Department of Scientific Research Management of Beijing Rehabilitation Hospital was responsible for overseeing the trial.

### Eligibility criteria {10}

The recruitment of PWP will be conducted through the outpatient clinic of Beijing Rehabilitation Hospital, Capital Medical University, as well as via the hospital website. Participants will be randomly assigned to either the tACS + MIRT group or sham-tACS + MIRT group in a 1:1 ratio, with 30 participants in each group. The trial group’s recruitment and intervention procedures will take place at Beijing Rehabilitation Hospital, Capital Medical University. Follow-up evaluations at each time point will be carried out either through an online follow-up system or within the outpatient clinic.

#### Inclusion criteria

- Conformity with the diagnostic criteria of idiopathic PD according to the 2015 Movement Disorder Society (MDS) criteria [3]
- Patients that are classified as 1–3 according to the Hoehn and Yahr scale [44]
- Age of 45–70 years and primary school education level and above
- The drug effect was stable for more than 2 weeks without adjustment and no DBS surgery was performed
- Those who can walk without assistance (including assistive devices) and need no help from others in daily life
- Those who voluntarily sign the informed consent form and confirm that they will be able to complete the treatment

#### Exclusion criteria

- The diagnosis is unclear or suspected of Parkinson’s syndrome (such as vascular, drug-induced, and post-infection Parkinson’s syndrome), multiple system atrophy, progressive supranuclear palsy, etc.
- Patients with cognitive, motor, speech, and other impairments caused by other nervous system diseases
- A history of epilepsy or other contraindications to tACS
- Subjects who are unwilling or unable to participate in MRI examinations (such as those with claustrophobia or non-MRI-compatible implants in the body)
- Serious concomitant diseases such as heart, lung, liver and kidney insufficiency
- Those who are participating in other clinical trials or are going to undergo DBS surgery or participate in other clinical trials in the next 12 months
- Patients who are unable to cooperate to complete the treatment or examination
- Patients who are not willing to sign the informed consent form

#### Dropping criteria

- Patients who revoke their informed consent
- Patients who discontinued treatment due to various factors

### Who will take informed consent? {26a}

Before the baseline assessment, all participants will be required to provide a written informed consent form. Participation will be voluntary, and patients may withdraw from the study at any time. Caregivers of potential participants will need to give informed consent prior to the allocation of time points. The informed consent process will ensure that participants and their caregivers are fully aware of the study’s objectives, activities involved, as well as its potential benefits and risks. Participants were encouraged to ask any questions they might have had during this process. Ultimately, it is up to the caregivers or participants themselves to decide whether or not they wish to participate in the study after being provided with comprehensive information. All data collected will be anonymized for confidentiality purposes. Multiple communication channels will be established in order to maintain close contact with participants.

### Additional consent provisions for collection and use of participant data and biological specimens {26b}

We will confirm with the participants if their data may be used.

## Interventions

### Explanation for the choice of comparators {6b}

The application of tACS as a non-invasive brain stimulation (NIBS) technique in clinical treatment is well-established. In this study, the inclusion of sham-tACS + MIRT in the control group served to elicit a placebo effect, with the only discernible distinction between the tACS + MIRT group and sham-tACS + MIRT being the presence or absence of electrical current. (Further details regarding this intervention can be found in the “Intervention description {11a}” section).

### Intervention description {11a}

After the initial baseline assessment (days 1–2), patients will be administered tACS + MITR or sham-tACS + MIRT for a consecutive period of 10 days (days 3–12).

#### tACS + MIRT group and sham-tACS + MIRT group

During each treatment session, patients will receive tACS or sham tACS stimulation for 40 min twice a day. The treatment duration was standardized for each day (40 min per session, twice daily for 10 days, totaling 20 sessions). Patients will receive a 10-day MIRT program based on tACS treatment, which includes four distinct rehabilitation exercises per day with each session lasting 30–60 min.

Participants were comfortably seated on reclining chairs to receive FDA-approved tACS from Nexalin Technology, Inc. The administration of tACS was carried out by trained medical professionals following standardized instructions. This trial utilized two tACS devices, one sham and one active, which were identical in terms of size, color, appearance, weight, and odor. Each participant was consistently assigned to the same device throughout the entire intervention.

Patients were instructed to consume water prior to the intervention, maintain a state of relaxation or even sleep, and minimize communication with medical personnel. Three Nexalin conductive electrodes were positioned above the head according to the 10/20 international placement system, with a 4.45 × 9.53 cm electrode placed on the forehead corresponding to Fpz, Fp1, and Fp2. Two 3.18 × 3.81 cm electrodes were placed on each side in the mastoid region. The tACS stimulation waveform consisted of ramp-up and ramp-down periods lasting for 180 s and 12 s respectively. It was a square wave with an average amplitude of 15 mA and was equally distributed from the frontal region to the mastoid areas (amplitudes are reported as zero-to-peak). All participants received a total of twenty sessions involving either true or sham stimulation at a frequency of 77.5 Hz and intensity of 15 mA, respectively. Sham tACS had no active stimulation.

#### MIRT procedure

I. Physical therapy. It involves warm-up activities followed by active and passive stretching and flexibility training. The physical therapist will conduct session in groups of four for a duration of 40 minutes.
II. Gait balance training. C-MiLL (Motek, Amsterdam/ Culemborg, Netherlands) and Balance Tutor (Meditouch, Netanya, Israel) will be utilized to enhance balance and gait. Patients will undergo 30-min training sessions once per day.
III. Aerobic training. Patients will engage in a 30-min aerobic workout using an upper and lower limb trainer (T5XR; Nustep, Ann Arbor, MI, USA).
IV. Speech therapy. The speech therapist will conduct one-hour group sessions of 4 patients.

Throughout the trial, we will have the option to modify parameters or discontinue treatment entirely in response to any adverse events that may arise. Physicians and therapists will document any such events in writing within the Electronic Data Capture (EDC) system for review by the study team. Additionally, falls, injuries, and other harmful incidents must be reported in accordance with regulations set forth by Beijing Rehabilitation Hospital affiliated with Capital Medical University.

### Criteria for discontinuing or modifying allocated interventions {11b}

The intervention will be terminated upon the patients’ request.

### Strategies to improve adherence to interventions {11c}

The participants will receive complimentary MIRT treatment, EEG, and MRI examinations to promote adherence to the interventions.

### Relevant concomitant care permitted or prohibited during the trial {11d}

Revised sentence: The concomitant therapies involve the administration of medications such as levodopa and benserazide, in addition to measures aimed at preventing complications. All other non-invasive or invasive brain stimulation interventions, including transcranial direct current stimulation or deep brain stimulation, will be strictly prohibited.

### Provisions for post-trial care {30}

Provisions for ancillary and post-trial care are not relevant to the study.

### Outcomes {12}

#### Primary outcome measures

The primary outcome indicator will be the PDQ-39, which assesses changes in quality of life from T0 to T2, T3, and T4. The PDQ-39 consists of 39 items that are categorized into eight domains: mobility, activities of daily living, emotional, well-being, stigma, social support, cognitions, communication, and bodily discomfort [46]. Patients were instructed to indicate the frequency of occurrence for each corresponding event within the preceding month. The final outcome of the scale encompasses a comprehensive index score, eight sub-core scores, and a weighted percentage reflecting problem severity. Higher scores (ranging from 0 to 100) indicate a greater burden on quality of life (QOL), with − 4.72 and + 4.22 serving as clinically significant thresholds for detecting improvements or deteriorations in QOL among individuals with PD [47]. The PDQ-39 has been translated and validated in multiple languages and cultural settings, making it a recommended tool by the Movement Disorders Society due to its proven effectiveness and stability [48].

#### Secondary outcome

The secondary outcome will encompass changes in motor symptom scales such as the third part of the Movement Disorders-Unified Parkinson’s Disease Rating Scale (MDS-UPDRS III) [49], Mini-balance evaluation systems test (Mini-BES Test) [50], and 6-min walking test (6MWT) [51] from T0 to T1, T2, T3, and T4. Additionally, alterations in non-motor symptom scales such as non-motor symptoms scale (NMSS) [52], Montreal cognitive assessment (MoCA) [53], and Hamilton depression scale (HAMD) [54] from T0 to T2, T3, and T4 will be evaluated.

- MDS-UPDRS III comprises of 18 items designed to evaluate motor function in individuals with Parkinson’s disease (PWP). The Chinese version of the MDS-UPDRS III demonstrates excellent reliability and validity, exhibiting a strong correlation with the conventional UPDRS [55].
- Mini-BES test is utilized for assessing gait and postural stability. This scale has demonstrated excellent inter-rater reliability (intraclass correlation coefficient (ICC) > 0.95) as well as high test-retest reliability (ICC > 0.95) [56].
- 6MWT measures the distance that a patient can quickly walk on a flat, hard surface within 6 min. We used the validated Chinese version which has shown a very high inter-rater correlation coefficient (ICC = 0:99) [57].
- NMSS allows quantitative evaluation of NMS of PD. The Chinese version of the NMSS can be considered a comprehensive, useful measure for NMS evaluation in Chinese PWP (Cronbach’s coefficient = 0.89, ICC = 0.99) [58].
- MoCA is a screening tool for mild cognitive impairment. The reliability of the Chinese version of MoCA has been proven in the clinic [59].
- HAMD will be utilized for the evaluation of a patient’s anxiety and depression levels. Its Chinese version has been empirically validated as a reliable and valid assessment tool [60].

#### Mechanism research

The changes in brain plasticity before and after rehabilitation intervention will be detected using Electroencephalogram (EEG) and fMRI.

##### Electrophysiological recordings

The EEG acquisition will be performed using a 64-lead EEG cap (model waveguard™ original 64, Netherlands) to facilitate real-time recording of EEG signals [61]. Electrodes will be positioned according to the international standard 10/20 system, with bilateral ear mastoids serving as reference electrodes. The sampling frequency will be set at 1000 Hz, and band-pass filtering between 1 and 30 Hz will be implemented. Additionally, efforts will be made to maintain electrode impedance below 10kΩ in order to minimize interferences during signal acquisition [62]. Subsequently, the acquired data will be stored in a computer for offline analysis.

##### fMRI protocol

Structural 3D T1-weighted and rs-fMRI data were collected using a 3.0t MRI scanner (GE SIGNA Pioneer) [63, 64]. The imaging dataset included T1-weighted, T2-weighted, fluid-attenuated inversion recovery (FLAIR), and T2-star weighted angiography magnetic resonance images. A total of 240 volumes of rs-fMRI data were acquired. Additional file 2 provides the parameter settings for each MRI sequence. Participants will be instructed to close their eyes and achieve a state of relaxation while maintaining wakefulness.

### Participant timeline {13}

The participant timeline is shown in Fig. 2.

### Sample size {14}

PASS11 software was used to calculate the sample size. According to the previous literature [65, 66], which includes the results of PDQ-39 in individuals with Parkinson’s disease, and in combination with this experiment, we designed a ratio of 1:1 between the tACS + MIRT group and Sham-tACS + MIRT group. The statistical power was set at 0.9, with a significance level (α) of 0.05. Based on these parameters, the required sample size for each group was calculated as 27. Considering an attrition rate of 10%, it was determined that each group would need a total of 30 patients, resulting in a combined total of 60 patients.

### Recruitment {15}

Recruitment commenced on June 1, 2023, and concluded on December 31, 2023, or upon reaching the required number of patients, whichever transpired first. In the event that there is an insufficient number of registrations prior to the deadline, we will submit an extension application. Participant recruitment encompassed two strategies: The initial step involves referring patients who visit the outpatient clinic at Beijing Rehabilitation Hospital to project researchers for recruitment purposes. The recruitment notices were additionally disseminated through the hospital’s official website and various other online platforms, facilitating interested patients to conveniently schedule appointments online. Patients with confirmed appointments underwent initial telephone screening conducted by a rehabilitation physician to assess their eligibility prior to undergoing on-site evaluation at the outpatient clinic. Ultimately, eligible participants who provided written informed consent were randomized and received treatment.

## Assignment of interventions: allocation

### Sequence generation {16a}

The randomization of patients in a 1:1 ratio will be conducted by an independent researcher who is not involved in the assessments or stimulation. This researcher will be solely responsible for dispensing randomization numbers to patients in the order of enrollment throughout the trial. We will utilize the complete randomization function of SPSS 27.0 statistical software (IBM, Chicago, IL, USA) to generate a table of random numbers, and the SPSS Visual Binning function has been integrated into its system. The process of randomization will be overseen by a blinded worker from DMC who will have exclusive authority over managing the electronic coding for assigning individuals. The process will ensure complete automation without any human intervention and will remain entirely undisclosed to both study investigators and prospective participants until the assignment of study groups. This document will be entrusted to Beijing Rehabilitation Hospital, Capital Medical University for secure storage.

### Concealment mechanism {16b}

The coding group for the assignment concealment process will be placed in a sealed envelope, which will be labeled with each participant’s code and securely held by the staff responsible for randomization. The envelopes will only be opened during tACS or sham-tACS sessions. To ensure proper blinding, participants will receive a password that will remain concealed during allocation by an independent staff member responsible for randomization. The tACS experimenter, who administers the intervention, will also remain unaware of the group assignments. Both the tACS and sham-tACS devices will have identical appearances and be marked as either A or B. Additionally, a DMC staff member responsible for randomization will inform the tACS experimenter to use either device A or B.

### Implementation {16c}

The sealed envelopes will be opened and resealed by the staff responsible for randomization prior to intervention. Subsequently, the tACS experimenter will be informed of the code.

## Assignment of interventions: blinding

### Who will be blinded {17a}

Both participants and clinic staff (including outcome assessors, caregivers, nurses, physical therapists, and statistical analysts) will remain blinded to group allocation until the completion of the study. Only the randomized investigators will have knowledge of the group assignments but will not disclose them to the patients. To ensure double-blinding, patients will be instructed not to discuss their treatment group with other patients or staff members. The disclosure of whether the intervention is tACS or sham-tACS will be withheld throughout the study.

### Procedure for unblinding if needed {17b}

When encountering serious adverse events that necessitate immediate unblinding, the scientific research management department head, data management department project leader, and statistician will jointly perform the unblinding process and meticulously document it. Unblinding will solely disclose the treatment received by a patient through a randomized number, without impacting the blinding of other participants. It will not be involved in efficacy analyses but will be included in safety analyses.

## Data collection and management

### Plans for assessment and collection of outcomes {18a}

The following data will be collected at baseline: gender, age, occupation, educational level, current medication status, and detailed physical and neurological examinations. Inclusion and exclusion criteria will be evaluated. PDQ-39 assessments will be conducted at T0, T2, T3, and T4. MDS-UPDRS III [49, 55], Mini-BES test [50, 56], 6MWT [51, 57], NMSS [52, 58], MoCA [53, 59], and HAMD, as well as other motor symptom scales and non-motor symptom scales, will be assessed at T0, T1, T2, T3, and T4 respectively. MRI and EEG scans will be evaluated at T0 and T1. Levodopa equivalent daily dose (LEDD) data will be collected at T0, T1, T2, T3, and T4. All these assessments will be conducted in a double-blind manner by raters who were unaware of the treatment administered to the patients. At T0 and T1, various safety indicators including blood routine, urine routine, stool routine, liver and kidney function tests, electrocardiogram readings, and other relevant parameters will be evaluated. Any adverse events occurring during the study will be documented. Participants will be asked for their consent regarding the use of their data if they choose to withdraw from the trial. Additionally, participants must obtain permission from the research team to share their pertinent information with university personnel involved in the study or relevant regulatory authorities. Prior to commencing the study, investigators will undergo standardized training on data collection strategies as well as guidelines for utilizing different scales to assess outcomes. Throughout the trial period, professionally trained researchers will collect data to ensure impartiality. In case subjects experience an “on–off” phenomenon during treatment response fluctuations; separate data collection sessions will take place during both “on” and “off” periods for subsequent comparison.

### Plans to promote participant retention and complete follow-up {18b}

To minimize dropout rates, participants will be reminded to attend all interventions and assessments. Participants and their caregivers will receive two telephone reminders (1 week and 1 day) prior to interventions and assessments. A dedicated hotline will be provided to offer assistance to participants and their caregivers regarding any inquiries or concerns.

### Data management {19}

Based on the clinical research data platform of Parkinson Medical Center, Beijing Rehabilitation Hospital, the EDC system will be utilized for data entry and management. The task of data entry will be performed by trained data collectors. Participant information will be securely stored in the EDC system, accessible to researchers through password authentication. Rigorous checks will ensure the completeness of case report forms and consistency between coding and subjects screened for enrollment. Data validation procedures will be implemented upon entry into the EDC system, with subsequent review of original cases based on these results. The final dataset will be exported as an Excel spreadsheet (Microsoft, Redmond, WA, USA). Fang Boyan is responsible for conducting interim analysis while making a final decision regarding trial suspension based on these interim results. Access to the finalized trial dataset will only be granted to authorized researchers.

### Confidentiality {27}

The privacy of all participants will be safeguarded by assigning their information a unique trial identification code, and the study data will be securely stored in a password-protected file accessible only to the data manager of the research team.

### Plans for collection, laboratory evaluation, and storage of biological specimens for genetic or molecular analysis in this trial/future use {33}

We will obtain informed consent from the patient and collect biological samples, including blood, urine, feces, and saliva, for molecular biology analysis at T0 and T1. A rigorous verification process with a double-signing system will be implemented for each sample to ensure timely acceptance and thorough examination. Prior to centrifugation, the staff will conduct a comprehensive review of the subject’s name, hospitalization number, bed number, and project details. Furthermore, specimens will be uniformly stored in standardized conditions at designated locations within clearly labeled specimen boxes.

## Statistical methods

### Statistical methods for primary and secondary outcomes {20a}

SPSS v27.0 software (SPSS Inc., Chicago, IL, USA) will be used for data processing and analysis. Demographic and baseline characteristics of the two groups will be described and analyzed to assess comparability. The PDQ-39 will serve as the primary outcome measure, and an analysis of covariance (ANOVA) model will be employed to estimate the adjusted means of patients’ quality-of-life scores (LSmeans), accounting for baseline differences, as well as calculate 95% confidence intervals for the difference in adjusted means. The count data in the secondary outcome indicators are described by frequency counts. The measurement data are tested for normality using the Shapiro–Wilk method and expressed as mean ± standard deviation if they follow a normal distribution. For non-normally distributed data, they will be described using (P25, P75). Between-group comparisons of motor function, non-motor function-related scale scores, and other secondary indicators between the two groups will be analyzed using appropriate methods based on the type of indicator. Component comparisons such as quantitative information will be analyzed using analysis of variance or Wilcoxon rank sum test. Categorical data will be analyzed by chi-square test or exact probability method, while hierarchical information will be assessed through rank sum Wilcoxon test or CMH test. The significance level for each test will be set at *P* < 0.05.

The MRI pre-processing will be conducted using the data processing assistant for Rs-fMRI, which operates in conjunction with the statistical parametric mapping software (SPM82). Subsequently, the EEG data will undergo offline processing through MATLAB software scripts and the EEGLAB toolbox.

### Interim analyses {21b}

No additional interim analyses will be conducted.

### Methods for additional analyses (e.g., subgroup analyses) {20b}

In case of baseline differences between the 2 groups (tACS + MIRT and sham-tACS + MIRT), statistical analyses will be adjusted to take into account specific variables that may have influenced the results for subgroup analysis (e.g., LEDD, illness duration, and age). The post hoc analyses will also be performed with Bonferroni corrected *t*-tests to examine the improvements in all outcome variables through comparisons across different assessment time points. Additionally, distinct exploratory analyses will be conducted for EEG and functional MRI data in order to gain a more comprehensive understanding of the impact of tACS intervention on brain plasticity.

### Methods in analysis to handle protocol non-adherence and any statistical methods to handle missing data {20c}

The intention-to-treat (ITT) analysis will be the primary analysis of this study, with per-protocol (PP) analysis as a secondary analysis in case patients were unable to receive the intervention due to issues related to the intervention itself, such as adverse effects, difficult administration, or adherence problems. Missing data will be handled using the last observation carried forward (LOCF) method. Sensitivity analyses will be conducted if there are imbalanced losses to follow up or excessive attrition.

### Plans to give access to the full protocol, participant-level data, and statistical code {31c}

Participant data is sensitive data and will be provided without participant identification upon reasonable request.

## Oversight and monitoring

### Composition of the coordinating center and trial steering committee {5d}

We have established an independent data and safety monitoring board (DSMB) for this study, which is not sponsored by any particular organization. The committee comprises a rehabilitation specialist, a neurologist, and a statistician who will monitor adherence to the trial design and standard guidelines.

### Composition of the data monitoring committee, its role and reporting structure {21a}

The trial design and adherence to standard guidelines will be overseen by the DSMB. This study, conducted at Beijing Rehabilitation Hospital, is a single-center investigation managed by the DSMB. A committee of rehabilitation specialists will be responsible for controlling the study protocol, while a neurologist will evaluate, manage, and classify all adverse events (AEs) that occur. Additionally, a statistician will review the data for safety purposes. It is important to note that DSMB members are independent of each other with no conflicts of interest.

### Adverse event reporting and harms {22}

The tACS procedure is considered safe, with no apparent short- or long-term harm observed. Potential low-grade adverse events may include seizures, headaches, site irritation pain, etc. However, post-treatment monitoring will be conducted using a safety questionnaire to assess any side effects.

All serious adverse events (SAEs) must be promptly reported within 24 h of occurrence to the principal investigator, ethics committee, and DSMB. Following thorough discussion and evaluation of these issues by the DSMB and principal investigators, they will have discretionary authority to determine whether discontinuation of the trial is necessary.

### Frequency and plans for auditing trial conduct {23}

The DSMB will convene regularly to conduct ongoing reviews of the trial’s progress, both at scheduled intervals every 3 months and throughout the duration of the trial. The DSMB is responsible for ensuring the timeliness, completeness, and accuracy of trial data, thereby necessitating investigators to promptly address any deficiencies or inaccuracies in their reporting. Additionally, a coordinating center will assume responsibility for managing day-to-day operations and providing comprehensive organizational support.

### Plans for communicating important protocol amendments to relevant parties (e.g., trial participants, ethical committees) {25}

Any modifications to the trial protocol must be communicated to and approved by the Ethics Committee of Beijing Rehabilitation Hospital before being implemented. In cases where important protocol modifications may affect the study’s conduct, they will also be reported to the trial registries.

### Dissemination plans {31a}

The results of this study will be published in peer-reviewed journals related to neuroscience and neurorehabilitation. The report will also be presented at national and international conferences and clinical forums and to other relevant health professionals and stakeholders, as well as to the participants. In addition, the included participants will receive a lay summary of their assessment reports after they finished the trial. A final report will be submitted to the trial register.

## Discussion

The aim of this study is to use tACS as an intervention tool for addressing abnormal oscillatory activity in PWP and enhancing overall function and quality of life by modulating neuroplasticity in ganglion-related brain regions. The presence of neural oscillations is linked to different behavioral states observed in PWP [67]. Deviation from physiological frequency bands serves as a distinctive feature of a broad range of pathologies known as thalamo-cortical dysrhythmias or oscillopathies [68]. Dopamine depletion leads to excessive synchronization of beta oscillations (15–30 Hz) in the basal ganglia and its related circuits [69]. Several studies have demonstrated the presence of finely-tuned, narrow-frequency gamma activity (referred to as fine-tuned gamma or FTG activity) in the spectra of local field potentials (LFPs) recorded from the globus pallidus interna (GPi), subthalamic nucleus (STN), and thalamus during rest conditions [70–72]. This activity exhibits contralateral enhancement during voluntary movements, thus exhibiting an opposite direction to beta-band activity. The use of tACS has shown its ability to induce neuroplasticity and regulate cortical function [22, 73].

TACS can effectively ameliorate motor retardation in PWP by regulating abnormal oscillations within the basal ganglia-thalamo-cortical network at the level of the primary motor cortex [27]. Gamma-tACS plays a crucial role in preparing and executing movements, and the improved motor function induced by gamma-tACS is associated with alterations in blood-oxygen-level dependent (BOLD) activity in M1. Furthermore, the effects of tACS extend beyond alterations in neural activity solely within the targeted region, encompassing functional connectivity and compensatory modulation occurring within task-related brain networks [74].

There is ongoing debate regarding the impact of weak currents applied to the scalp on neural activity, as studies have shown that scalp currents are attenuated by approximately 75% due to soft tissues and the skull. Low-intensity current stimulation is typically employed for localized and superficial dermal stimulation [75–78]. Conversely, higher-intensity AC stimulation exhibits a more pronounced effect in entraining neural oscillations and influencing neural circuits. Notably, one study demonstrated a convincing induction effect on brain networks through strong AC stimulation administered to anesthetized patients via intracranial screw electrodes [79]. Measurements conducted on rodents and human cadaver brains have revealed that scalp-applied stimulation necessitates a minimum current exceeding 6 mA to transiently and efficiently alter brain networks; however, Hi-tACS remains underutilized in PWP [77]. However, a tACS study utilizing a frequency of 77.5 Hz did not yield significant improvements in motor and psychological symptoms among PWP when compared to a placebo intervention [80]. We have identified several potential reasons for these findings. Firstly, the previous trial only administered tACS treatments 10 times per patient, which may have resulted in an insufficient cumulative dosage that contributed to the ultimately negative outcome. In our experiment, each patient received 2 daily sessions of tACS over a period of 10 days, totaling 20 treatment sessions, thereby increasing the dosage in order to achieve more favorable results. Additionally, the prior investigation had a limited sample size of only 23 participants for their simple tACS study, potentially influencing the negative outcomes observed. To address this limitation and provide stronger data support for our trial, we aimed to recruit 60 participants and expand the sample size accordingly.

The previous trials have primarily focused on the administration of tACS alone, whereas our trial integrates tACS with MIRT based on a “central-peripheral-central” model. In this novel rehabilitation model, positive feedback is utilized to enhance brain plasticity and the rehabilitative effects following brain injury. MIRT is a goal-directed rehabilitation program designed to address functional impairments caused by Parkinson’s disease through explicit and implicit learning strategies [40]. Randomized controlled studies have demonstrated that MIRT significantly improves the quality of life in PWP [81]. Finally, the previous study solely compared behavioral outcomes pre- and post-treatment of subjects. We have previously conducted a study on 4 Hz tACS in PD using cortico-subcortical co-activation pattern (CAP) analysis on rs-fMRI data, which demonstrated that cortical and subcortical regions exhibit co-activation during the resting state and form distinct CAPs. Moreover, the occurrence of cortical and subcortical CAPs can be enhanced following tACS treatment [82]. This alteration in brain function may precede the observed behavioral changes. Therefore, we have also incorporated EEG and fMRI examinations to assess brain network function in this study.

Through a randomized double-blind design, this trial will validate the efficacy and long-term effects of tACS on quality of life and overall functioning among PWP who receive true tACS or sham tACS. The ultimate goal is to establish a correlation between the behavioral improvements induced by tACS in PWP and changes in the functional connectivity of neural networks, as well as biomarkers of neurobiological plasticity using structural and functional magnetic resonance imaging techniques. Additionally, we aim to investigate the interaction between the stimulated area’s functional connectivity and other brain regions within the executive function system. This study also seeks to examine the relationship between baseline neural circuits in the brain and those after tACS intervention, particularly focusing on any effects on emotional and motor circuits. By applying a novel rehabilitation program combining tACS with MIRT for PWP, our results can contribute towards establishing a standardized model for PD rehabilitation that will provide evidence-based medical support for enhancing rehabilitation treatment models and improving the quality of life for PWP.

## Trial status

The study is currently ongoing at the time of submitting this manuscript (December 2023), utilizing protocol version 2. The protocol has undergone review by the ethics committee and has been uploaded to the web for accurate documentation purposes. The initial patient was enrolled in October 2023, and this trial is projected to conclude by the end of 2024.

### Supplementary Information

**Additional file 1.** SPIRIT 2013 checklist.

**Additional file 2.** The parameter settings of each MRI sequence.

## Data Availability

The datasets generated and analyzed during the current study will be available from the corresponding author on reasonable request.

## Abbreviations

CAP: Co-activation pattern
DBS: Deep brain stimulation
DSMB: Data and safety monitoring board
EDC: Electronic data capture
EEG: Electroencephalogram
fMRI: Functional magnetic resonance imaging
HAMD: Hamilton depression scale
Hi-tACS: High-intensity transcranial alternating current stimulation
LEDD: Levodopa equivalent daily dose
MDS-UPDRS: Movement Disorders–Unified Parkinson’s Disease Rating Scale
MIRT: Multidisciplinary intensive rehabilitation treatment
Mini-BES Test: Mini-balance evaluation systems test
MoCA: Montreal cognitive assessment
NIBS: Non-invasive brain stimulation
NMSS: Non-motor symptoms scale
QOL: Quality of life
RCT: Randomized controlled trial
tACS: Transcranial alternating current stimulation
PDQ-39: 39-Item Parkinson’s disease questionnaire
PWP: Patients with Parkinson’s disease
6MWT: 6-Min walking test
