# Supplementary material for: Transcranial alternating current stimulation improves quality of life in Parkinson’s disease: study protocol for a randomized, double-blind, controlled trial
Source: Trials. 2024 Mar 20;25:200. doi: 10.1186/s13063-024-08045-5 (PMC10953283; doi:10.1186/s13063-024-08045-5)
Supplement: Supplementary file 2 — Additional file 2. The parameter settings of each MRI sequence. [file 13063_2024_8045_MOESM2_ESM.docx]

## Additional File 2. The parameter settings of each MRI sequence

| T1-weighted MR images | repetition time (TR) = 500 ms, echo time (TE) = 10 ms, flip angle = 90°, field of view = 240 × 240 mm^2^, matrix size = 320 × 256 pixels, slice number/thickness = 20/5.0 mm |
| --- | --- |
| T2-weighted MR images | TR = 4500 ms, TE = 100 ms, flip angle = 90°, field of view = 240 × 240 mm^2^, matrix size = 320 × 256 pixels, slice number/thickness = 20/5.0 mm |
| Fluid attenuated inversion recovery (FLAIR) MR images | TR = 15000 ms, TE = 100 ms, inversion time (TI) = 3000 ms, flip angle = 90°, field of view = 240 × 240 mm^2^, matrix size = 320 × 256 pixels, slice number/thickness = 20/5.0 mm |
| T2-star weighted angiography (SWAN) MR images | TR = 76.6–84.80 ms, TE = 41.82–45.00 ms, flip angle = 15°, field of view = 240 × 240 mm^2^, matrix size = 384 × 320 pixels, slice number/thickness = 76/2.0 mm. (The minimum TR and TE values were automatically selected according to the patient's weight) |
| Rs‐fMRI images | TR = 2000 ms, TE = 35 ms, flip angle (FA) = 90°, field of view (FOV) = 280 × 280 mm^2^, matrix resolution =128 × 128, slice thickness = 4 mm, and number of slices = 40. |

Magnetic resonance imaging data containing T1-weighted magnetic resonance images, T2-weighted magnetic resonance images, fluid attenuated inversion recovery magnetic resonance images, and T2-star weighted angiography magnetic resonance images were acquired from a GE SIGNA Pioneer 3.0-Tesla scanner.And a total of 240 volumes of rs‐fMRI data were collected. The parameter settings of each magnetic resonance imaging sequence were listed in Supplementary Table 1.
